# *SBEMimage*: Versatile Acquisition Control Software for Serial Block-Face Electron Microscopy

**DOI:** 10.3389/fncir.2018.00054

**Published:** 2018-07-31

**Authors:** Benjamin Titze, Christel Genoud, Rainer W. Friedrich

**Affiliations:** ^1^Friedrich Miescher Institute for Biomedical Research, Basel, Switzerland; ^2^Faculty of Natural Sciences, University of Basel, Basel, Switzerland

**Keywords:** SBEM, imaging software, connectomics, serial block-face, SEM, volume EM, microtome, 3View

## Abstract

We present *SBEMimage*, an open-source Python-based application to operate serial block-face electron microscopy (SBEM) systems. *SBEMimage* is designed for complex, challenging acquisition tasks, such as large-scale volume imaging of neuronal tissue or other biological ultrastructure. Advanced monitoring, process control, and error handling capabilities improve reliability, speed, and quality of acquisitions. Debris detection, autofocus, real-time image inspection, and various other quality control features minimize the risk of data loss during long-term acquisitions. Adaptive tile selection allows for efficient imaging of large tissue volumes of arbitrary shape. The software’s graphical user interface is optimized for remote operation. In its user-friendly viewport, tile grids covering the region of interest to be acquired are overlaid on previously acquired overview images of the sample surface. Images from other sources, e.g., light microscopes, can be imported and superimposed. *SBEMimage* complements existing *DigitalMicrograph (Gatan Microscopy Suite)* installations on 3View systems but permits higher acquisition rates by interacting directly with the microscope’s control software. Its modular architecture and the use of Python/PyQt make *SBEMimage* highly customizable and extensible, which allows for fast prototyping and will permit adaptation to a wide range of SBEM systems and applications.

## Introduction

The efficient reconstruction of neuronal circuits and other biological ultrastructure by electron microscopy requires fast, reliable, and high-quality acquisition of large volumetric image datasets (Lichtman and Denk, 2011; Denk et al., 2012). Several automated acquisition methods based on scanning or transmission electron microscopy have been developed for this purpose (reviewed in Briggman and Bock, 2012; Peddie and Collinson, 2014; Titze and Genoud, 2016). One approach is to collect series of ultrathin sections on a supporting structure before image acquisition (Hayworth et al., 2006; Schalek et al., 2012), which preserves sections but requires solutions for large-scale section collection and image alignment. Alternatively, a stained and embedded tissue block can be cut with an ultramicrotome inside the vacuum chamber of a scanning electron microscope that is used to image the block-face after each cut (Denk and Horstmann, 2004). This approach, termed serial block-face electron microscopy (SBEM), achieves reliable thin sectioning and requires only minimal alignment of successive images. However, it does not permit repeated imaging of the same tissue since sections are destroyed during the acquisition. It is therefore crucial to ensure high reliability of data acquisition and to have efficient error detection procedures in place to prevent data loss when running long SBEM acquisitions. Also, to minimize the duration of such acquisitions, it is desirable to efficiently restrict image acquisition to regions of interest. Furthermore, solutions to integrate data acquisition with image post-processing would allow to perform operations such as stitching, alignment, and image analysis while the acquisition is running.

SBEM has been successfully used for ultrastructural imaging of large tissue volumes in the past (Briggman et al., 2011; Wanner et al., 2016a,b; Schmidt et al., 2017, among others), but further software improvements are desired to optimize the acquisition workflow. After the initial development of automated SBEM (Denk and Horstmann, 2004), a commercial system (Gatan 3View) based on the original design was introduced and is now widely used. The 3View microtome and image acquisition process are controlled via the proprietary software *DigitalMicrograph* (*Gatan Microscopy Suite; GMS 2* or *3*). This software provides the basic functionality to set up and run acquisitions on 3View systems. However, it does not provide key features such as automatic debris detection that are important to prevent data loss during long acquisitions, and it does not allow for fine-grained control and customization. Furthermore, *DigitalMicrograph* currently limits the image acquisition rate to 2 MHz. These restrictions prompted the development of *SBEMimage* as a flexible and powerful open-source acquisition platform for SBEM/3View systems.

*SBEMimage* provides essential quality control features for long-term acquisitions. When running an acquisition continuously over days, weeks or even months, the following problems can occur: (1) debris falling on the sample surface and obscuring the region of interest; (2) focus and astigmatism drifts or jumps; (3) electron beam instability; (4) malfunctions of the (proprietary) control software (here: *DigitalMicrograph* and *SmartSEM*); (5) I/O errors such as disk or network drive unavailability for writing image data; (6) hardware failures (stage motors, SEM cathode, backscattered electron detector, vacuum system, power). With these issues in mind we designed *SBEMimage* to provide improved error handling capabilities, more extensive monitoring functions, and other features that enhance stability and reduce user interaction time. An automatic debris detection and removal mechanism and a reliable autofocus function combined with slice-by-slice tile monitoring solve problems (1), (2), and (3). Error detection procedures pause the acquisition when errors of type (4), (5), or (6) occur that cannot be resolved by the software. Additional features allow for imaging of volumes with complex geometries and for the integration of image analysis procedures into the acquisition process. A flexible and user-friendly graphical interface minimizes the risk of human error when setting up and running acquisitions. The highest priority during development was to prevent data loss in case of major failures.

*SBEMimage* is an open-source project, released on GitHub under the MIT License, and intended as a free-to-use community-supported resource. The code repository, installation instructions, and further documentation can be found on GitHub^1^. The software was developed with a modular architecture, and the source code is fully commented, which should allow Python programmers to easily customize it and add new functionality.

## Implementation

**FIGURE 1:**
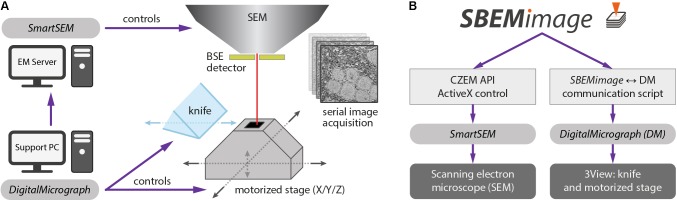
**(A)** Conventional setup of the 3View system. The software *SmartSEM* (Carl Zeiss Microscopy, Cambridge, United Kingdom), running on the EM Server PC, controls the scanning electron microscope (SEM). The software *DigitalMicrograph* (Gatan), running on a support PC, controls the 3View hardware (diamond knife and motorized stage). *DigitalMicrograph* can also indirectly control the SEM via *SmartSEM*. **(B)** *SBEMimage* interacts with *SmartSEM* through a proprietary remote API, provided by Carl Zeiss Microscopy, and with *DigitalMicrograph* through a custom-written communication script. *SBEMimage* can thus control all relevant low-level functions of the SBEM system and exert full control over the acquisition process.

We implemented *SBEMimage* in Python (version 3.6), a high-level programming language that is widely used in the scientific community. The toolkit PyQt 5 was used for the graphical user interface. *SBEMimage* is currently designed to operate a 3View microtome combined with a ZEISS Merlin microscope. It interacts with two pieces of existing proprietary software: (1) The microscope control software *SmartSEM*, which must be installed on the EM server PC, and (2) *DigitalMicrograph* (*Gatan Microscopy Suite* 2 or 3), which runs on a support PC. Together, these two applications control the SBEM system in the conventional configuration (**Figure 1A**).

Acquiring images in *DigitalMicrograph* with *DigiScan* (Gatan’s scan generator) limits the acquisition rate to 2 MHz. To overcome this limit, *SBEMimage* acquires images via *SmartSEM*, which permits acquisition rates of up to 40 MHz. This approach requires an adapter that connects the amplified BSE detector output to one of the microscope’s acquisition boards. Details are provided on the GitHub page.

To achieve maximum flexibility, we first had to find a way to control all relevant low-level functions of the 3View system through Python commands. For the ZEISS Merlin and other ZEISS microscopes, a powerful API already exists: the *SmartSEM* Remote API, developed by Carl Zeiss Microscopy, implemented as an ActiveX control, with test programs available in C++, C#, and Visual Basic. We built a wrapper module that allows all relevant commands of that API to be used in a Python application. This module may be adapted in the future to allow SEMs from other manufacturers to be controlled with the same *SBEMimage* Python commands.

*DigitalMicrograph* offers an internal scripting language that provides a number of commands to control the 3View stage and the microtome’s knife. However, there is no publicly accessible and documented API that lets programs running outside of *DigitalMicrograph* use these functions. Our solution was to create a communication script that runs in *DigitalMicrograph* and enables the exchange of commands and parameters with external programs. This information exchange is achieved through reading and writing files. In 0.1-s intervals, the *DigitalMicrograph* script checks for the existence of a trigger file. If this trigger file is detected, the script reads an input file that contains a command and up to two parameters. In this way, *SBEMimage* can send commands to *DigitalMicrograph* and read return values. Through the *SmartSEM* remote API and the *DigitalMicrograph* communication script, *SBEMimage* can thus control all relevant low-level functions of the SBEM system (**Figure 1B**).

## Description of the User Interface

**FIGURE 2:**
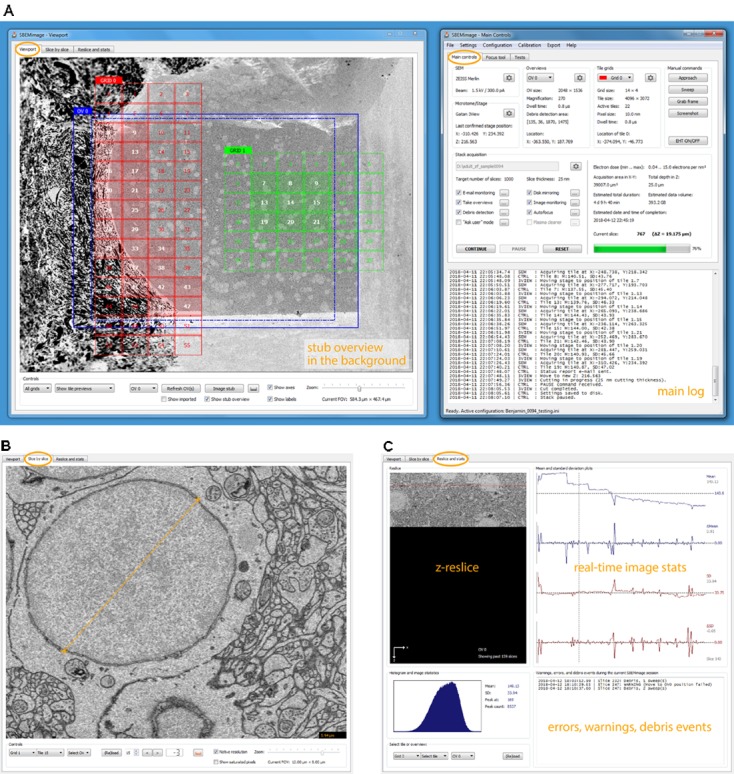
*SBEMimage* user interface. **(A)** The default window arrangement during a stack acquisition. Recommended screen resolution is 1920 × 1080. Left window: Viewport showing an overview image and two tile grids. The highlighted tiles have been selected for imaging. A low-resolution stub overview mosaic is displayed in the background. The right window contains the main controls: Settings are shown for the SEM and the microtome, the current overview image and one of the tile grids. In the lower part of the window, all activity is logged. In the stack acquisition panel, users can select or deselect features to be used during the acquisition and watch the progress of the acquisition. Electron dose range and duration/storage estimates are shown on the right side of the panel. **(B)** The slice-by-slice viewer lets the user view recently acquired images at full resolution. With the mouse wheel, the user can scroll through consecutive slices to assess cutting and image quality. **(C)** In the statistics tab, the user can select an overview image or a tile, for which the current reslice, the histogram, and mean/SD plots are shown. All errors, warnings, and debris events are logged in the lower right part of this tab.

The graphical user interface consists of two windows, by default positioned next to each other on a wide screen (**Figure 2A**). The interface was designed with remote desktop software such as *TeamViewer* and *VNC* in mind: All functions (including basic SEM operations such as turning on the high voltage and focusing the beam) are accessible on a single screen.

The window “Main Controls” on the right displays at a glance all relevant settings, the acquisition status, the current electron dose, and real-time estimates for the duration of the acquisition and the storage size of the dataset. Various dialogs let the user set up all acquisition parameters and, when using the program for the first time, perform a motor speed and stage calibration. Calibrating the stage is necessary because the *X* and *Y* motor axes are rotated and scaled with respect to the SEM coordinate system (**Supplementary Figure S1B**). Additional tabs of the “Main Controls” window contain a novel tool for manual focusing (explanation below) and various functions for testing and debugging.

The window “Viewport” on the left lets the user set up and monitor acquisitions visually. The workspace shown in the viewport’s main tab covers the entire accessible range of the stage motors. When sufficiently zoomed out, the stage boundaries are shown as solid white lines, and the *X* and *Y* stage axes as dashed white lines. To obtain an overview of the entire surface of the sample holder (“stub”) mounted on the 3View stage, the user can acquire a “stub overview image,” a large low-resolution (372 nm pixel size) mosaic of specified size that is displayed in the workspace as the main background image (**Supplementary Figure S1A**).

The user can use this stub overview image to comfortably locate the region of interest and navigate there to acquire smaller overview images at higher resolution (typically 100–200 nm pixel size). To acquire image tiles at the target resolution for analysis (typically 5–20 nm pixel size), the user can set up a tile grid in the region of interest. Grid size, tile size, overlaps/gaps between tiles, and acquisition parameters (frame size, pixel size, and dwell time) are specified for each grid. Tiles can be individually selected or deselected for imaging and the whole grid can be shifted when necessary. For complex acquisition tasks, multiple overviews can be set up to cover the region(s) of interest, and multiple grids can be created with different imaging parameters. The user can choose for each overview image and for each grid whether it should be acquired on every slice, or in intervals. This permits, for example, to image a region of interest with alternating pixel sizes from one slice to the next, or to acquire an overview stack at low resolution with a high-resolution mosaic on every tenth slice.

The basic elements described above are displayed as different layers inside the viewport. The background layer consists of the stub overview image, which provides the main reference frame for an acquisition. The layer above contains the overview images that cover the regions of interest. They are primarily used for debris detection and to position the tile grids. The tile grids are placed in the next layer above the overview images. Finally, additional imported images (see feature description below) are shown in the foreground, typically with a transparency setting that allows the user to see through these images. Users can freely position all tile grids, overviews, and imported images within the accessible motor range and choose whether to show or hide them.

The visual scene can be panned by left-click dragging, and zoomed in and out with the mouse wheel or a zoom slider. All elements can be selected and edited with mouse clicks and dragged to new positions. The viewport is fully functional even while an acquisition is running.

## Brief Description of Features

**FIGURE 3:**
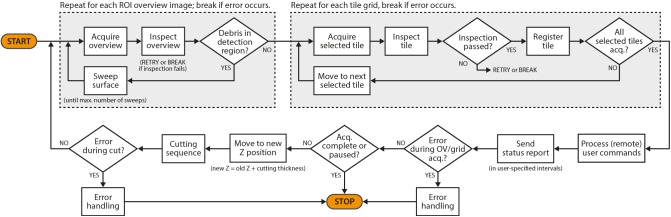
A simplified flowchart of *SBEMimage*’s stack acquisition cycle.

In the following, we have summarized the key features of *SBEMimage* and a few additional features that may be of interest for special applications. Users can select before and during an acquisition which features should be active while the acquisition cycle (**Figure 3**) is running.

### Debris Detection and Removal

When the debris detection option is activated, each newly acquired overview image is compared to the one on the previous slice to check if debris is present. To initiate this process, the user is asked to confirm that the very first overview image at the beginning of a stack does not contain debris. Three different methods (based on comparisons of mean/SD values, histograms, and pixel differences, respectively; for details, refer to the GitHub repository) are currently available to detect debris on the surface. The default detection procedure divides the region of interest into four quadrants and compares the pixel mean and standard deviation in each quadrant between successive images. If the maximum difference across all four quadrants exceeds a user-defined threshold, the program assumes that debris is present. In this case, *SBEMimage* performs a “sweep” (first described in Helmstaedter et al., 2013): After the sample is lowered by 70 nm (or a different amount as specified by the user), the knife is moved across the sample surface in an attempt to push the debris away. The process can be repeated until the user-specified maximum number of sweeps has been reached. Depending on the option settings, the program will then either pause the acquisition or continue regardless of the debris. Detection parameters should be fine-tuned for a given sample and imaging settings to achieve optimal results. In our hands, the automated procedure detected and removed all medium- to large-size chunks of debris (>10 μm). The detection and removal of very small flakes of debris on the order of microns or smaller may, however, be less efficient, depending on sample properties, imaging parameters, and residual charging.

### Focus and Stigmation Control

**FIGURE 4:**
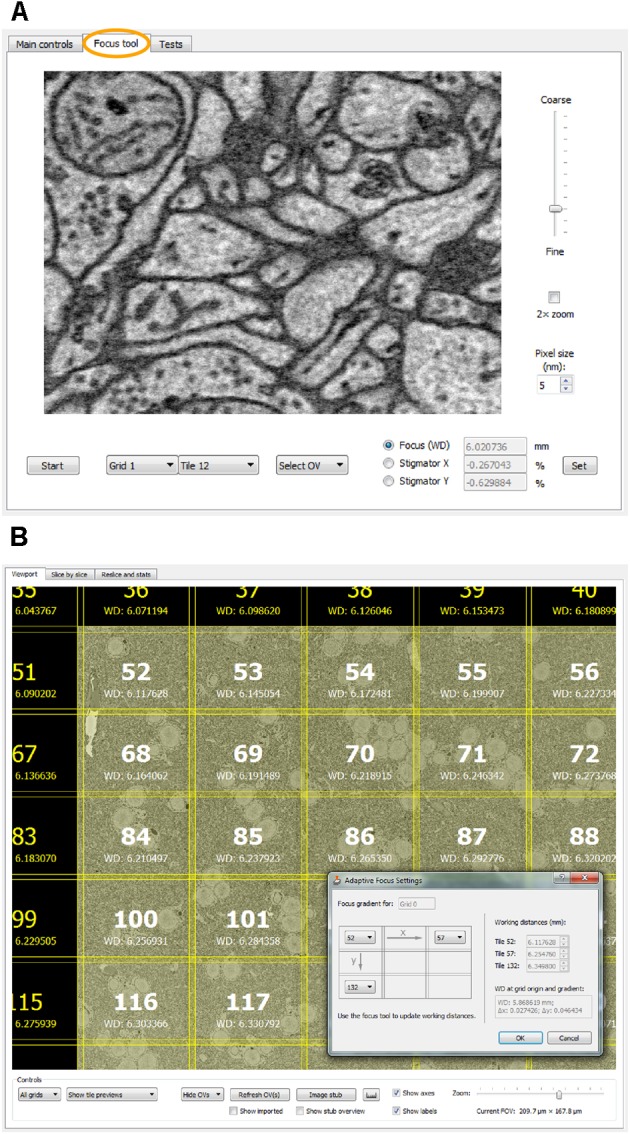
**(A)** The focus tool (the second tab in the main controls window) allows the user to remotely focus individual tiles or overview images and adjust the stigmator settings. A brief explanation is provided in the main text. **(B)** For imaging tilted surfaces, *SBEMimage* offers a focus gradient correction feature. When the optimal focus for three tiles on a given grid is specified (see drawing in the dialog window), *SBEMimage* computes the gradient and adjusts the focus (working distance) automatically for every tile in the grid. In the viewport, the different working distances for each tile are displayed below the tile number.

For remote focusing with *TeamViewer* or *VNC*, *SBEMimage* offers a **focus tool** that acquires a 512-pixel × 384-pixel through-focus series on a specified tile or overview image (**Figure 4A**). The user can then choose the optimal focus setting from this image series. Optimal X/Y stigmation settings are chosen with the same procedure. For refinement, the cycle can be repeated. This focus tool offers two advantages: (1) It can be used remotely (when no adjustment knobs and no access to the EM server PC are available) and even at slow connection speeds of the remote desktop software; (2) The procedure minimizes the electron dose, at least in case of small focus corrections, since each image series is acquired only once, whereas manual refocusing with the microscope software relies on continuous scanning. The focus tool can also be used to set working distances for individual tiles, which is needed for setting up an **adaptive focus for tilted surfaces**. A slight tilt of the 3View knife is usually unavoidable, which for very large distances across the sample surface can lead to a difference in working distance: One part of the region of interest may be well focused, but a more distant part will be out of focus. This also typically occurs when mounting a sample in the 3View holder that will not be cut. Any initial tilt of the surface will thus not be physically corrected by the blade. For these cases, *SBEMimage* offers a gradient correction mechanism (**Figure 4B**). To correct the focus during an acquisition, two **autofocus** methods are implemented in *SBEMimage*: Method (1) uses the built-in *SmartSEM* autofocus/stigmator, which is called in regular intervals, as specified by the user. The user can choose the reference position(s) where the focus/stigmation procedure should be performed, and decide if both autofocus and autostigmation are to be performed on the same slice or a specified number of slices spaced apart to minimize beam exposure. Method (2) uses a continuous heuristic autofocus procedure based on autocorrelation. The algorithm works on tiles that have already been acquired and applies corrections continuously. This approach was first used in Briggman et al. (2011) and is described in appendix A of Binding et al. (2013).

### Adaptive Tile Selection

**FIGURE 5:**
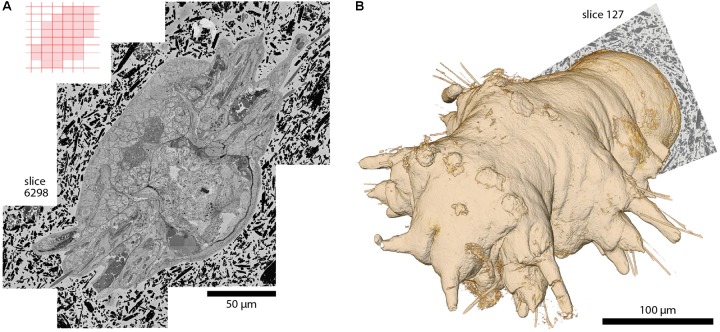
A whole *Platynereis dumerilii*, late nectochaete stage (6 days post-fertilization), acquired with *SBEMimage*. Imaged with a ZEISS Merlin SEM at 1.8 keV landing energy, 270 pA beam current, and 0.8 μs dwell time. 11,416 slices with >200,000 image tiles at 10 nm × 10 nm × 25 nm voxel size. *Platynereis* were provided by H. Martínez and D. Arendt, EMBL, Heidelberg. Sample preparation by P. Machado and Y. Schwab, EMBL, Heidelberg. Alignment: A. Wanner. Silver epoxy embedding was used to reduce charging, as described in Wanner et al. (2016b). **(A)** Stitched mosaic, slice 6298. The corresponding tile pattern is shown in the upper left corner. Adaptive tile selection was used throughout the stack acquisition to efficiently capture the irregular shape of the worm along its (tilted) anterior-posterior axis. **(B)** The worm’s 3D shape, as reconstructed from the EM dataset. Segmentation and 3D rendering: www.ariadne.ai.

Within each grid, tiles can be selected as “active,” or deselected, with single mouse clicks. Only active tiles are acquired. Deselecting tiles outside the region of interest therefore makes imaging non-rectangular regions of interest more efficient (**Figure 5A**). When debris detection is used, *SBEMimage* can automatically adjust the detection region to cover only the region of the overview image that contains active tiles. The active tile pattern can be adjusted by the user while an acquisition is running. Grids can be shifted when the acquisition is paused. When an error occurs or the user pauses the acquisition, *SBEMimage* remembers which tiles on the current slice have already been acquired and will resume the acquisition at the correct position in the grid.

### Slice-by-Slice Tile Monitoring

On each slice, the mean and standard deviation of a selected tile is compared to the mean and standard deviation of the same tile in the previous slice. If the differences exceed user-defined thresholds, the acquisition is paused. This approach permits detection of sudden unexpected changes (loss of focus, darkening, and blank images). In the second tab of the viewport window (**slice-by-slice viewer**), the user can load the most recently acquired tiles and overviews into memory and view them at full resolution (**Figure 2B**). Panning and zooming work as in the viewport. The mouse wheel and control buttons let the user go backward and forward through the acquired slices to check if the cutting is regular and to assess image quality. Distances in the images can be measured with a ruler tool (also available in the viewport). The viewport offers ***in situ* tile previews** during acquisitions. A preview image (512 pixels × 384 pixels) is generated from each tile as soon as it is acquired. This preview image is then immediately displayed in the tile grid, such that the user can inspect it in its relative position to the other tiles. To check whether the specified overlaps are sufficient and the stage calibration is accurate, tile previews can be shown either in “overlap mode,” where they are placed at the exact stage positions where they were acquired, or in “gap mode,” where artificial gaps are put between the tiles, which is useful to verify the alignment and the width of overlapping areas.

### Image Inspection and Selection

After each image acquisition, the module *ImageInspector* reads the acquired image file from disk into memory and performs a number of image integrity and quality checks. If a check fails, the user is alerted via e-mail and the acquisition is paused automatically. It is also possible to test incoming images for other user-defined features and either select or discard them on the basis of these tests. This feature can save storage space and also time since it can be exploited to carry out some data processing and pre-selection operations already during the acquisition.

### Data and Metadata Handling

Images are first saved on a primary drive (usually the local hard disk or SSD of the support PC; see **Supplementary Figure S2** for *SBEMimage’s* folder structure and file name conventions). The user can specify a second local or network drive as a **mirror drive**, where the acquired image data and the metadata are mirrored during the acquisition. This feature provides a backup solution and makes the data available for post-processing while the acquisition is running. For seamless integration into an image post-processing pipeline, *SBEMimage* **metadata** can be exported in the *TrakEM2* image list format (Cardona et al., 2012). Metadata can also be transferred to a remote server while the acquisition is running. *SBEMimage* integrates with the *Volume Image Environment* (VIME; Gerhard et al., in preparation^2^), which allows for the visualization and post-processing of images in real-time as they are acquired by *SBEMimage*. A communication protocol between *SBEMimage* and *VIME* lets users implement a flexible remote quality control system. In case of problems with post-processing of the acquired image data – for example when stitching is not possible due to insufficient overlap – the acquisition can be paused remotely by *VIME* and the user is notified.

### Error Handling

When an error is detected, *SBEMimage* in most cases makes a second or third attempt to carry out the failed operation and writes a warning message into the log. If the second/third attempt also fails, the acquisition is paused and the user is notified via e-mail. Error codes have three digits and are grouped according to the first digit: (1) errors related to communication with *DigitalMicrograph*; (2) errors related to 3View/SBEM hardware; (3) errors related to SmartSEM/SEM; (4) I/O errors; (5) errors related to the acquisition process and image inspection; (6) user-defined errors. Detailed information about the entire acquisition process including all warnings and errors is saved in the main log.

### Additional Features

#### Configurations for Multi-User Multi-Project Environments

All system settings, calibrations, acquisition parameters, and workspace options are stored in configuration files. This allows each user to maintain his or her own configuration and to work on different projects on the same system.

#### E-Mail Monitoring and Control

In user-specified intervals, a status report is sent via e-mail. The user can customize the content of the report (screenshots, log files, images, and reslices). When a critical error occurs, *SBEMimage* immediately sends an e-mail to alert the user. The user can also send commands to *SBEMimage* via e-mail, for example to pause an acquisition remotely when *TeamViewer* or *VNC* are unavailable.

#### Importing Overview Images

Existing images can be imported into the workspace at a variable pixel size, stage position, rotation angle, and transparency, which is of special interest for correlative light and electron microscopy (CLEM). For example, light microscopic images can be loaded and superimposed on EM images of the same tissue to align cell bodies or other structures.

#### Real-Time Reslices, Histograms, and Statistics

The *z*-reslice image for a user-selected overview or tile, its histogram, and its time course of mean and standard deviation measurements are shown in the third tab of the viewport window (**Figure 2C**). By clicking on the plots, the user can select a past slice along the time axis, for which the histogram and mean/SD values will be displayed.

#### Plasma Cleaner Control

*SBEMimage* includes a module to control the downstream asher GV10x (ibss Group Inc., Burlingame, CA, United States) to clean the inside of the microscope’s vacuum chamber and the surface of the BSE detector.

## Summary and Outlook

With the development of *SBEMimage* we have addressed key problems encountered frequently in SBEM acquisition projects. The software has been tested extensively and used in multiple projects including long-term acquisitions. One example is a 6-week acquisition of an image stack covering an entire specimen of *Platynereis dumerilii* at a voxel size of 10 nm × 10 nm × 25 nm (**Figure 5**). This dataset comprises >200,000 image tiles that were unevenly distributed over 11,416 slices (approximately 18 tiles per slice on average). The efficient acquisition of this dataset depended critically on two *SBEMimage* features, debris detection and adaptive tiling. Debris was detected and removed on 493 slices. Hence, a substantial fraction of slices would have been compromised at least locally without automated debris detection. Adaptive tile selection allowed us to acquire data only from those tiles that contained sample tissue. The entire *Platynereis* specimen was contained within a bounding cuboid of approximately 275 μm × 260 μm × 285 μm. However, because the specimen was tilted and had a complex shape, image data had to be acquired from only ∼30% of this cuboid. Adaptive tiling therefore saved a substantial amount of time and resources for data acquisition, post-processing, and storage.

The release of *SBEMimage* on GitHub^3^ allows potential users to test it on their systems and adapt it to their needs. The software currently supports Gatan 3View microtomes and ZEISS SEMs. Operation of *SBEMimage* with devices from other manufacturers will require the adaptation of *SBEMimage* modules with device-specific code for low-level functions. The development of *SBEMimage* was inspired by a previous open-source microscopy project that has been highly successful: *ScanImage,* a widely used application for operating laser scanning microscopes (Pologruto et al., 2003).

A promising future application of *SBEMimage* are “data-driven acquisitions,” where image data is used in real-time by the program to algorithmically determine what to acquire next. *SBEMimage* offers an ideal framework to implement such an approach. For example, machine learning could be used to detect tissue boundaries in overview images. For a given volume to be acquired, the user would specify the starting grid configuration and select all the tiles needed to cover the tissue. The algorithm would then decide from slice to slice whether to shift or expand the tile pattern to follow the tissue through the sample.

Another potential application is sparse and selective imaging of tissue volumes. For many SBEM applications, only a fraction of the acquired data is actually needed for further analysis. As described above, substantial time can be saved by adaptive tile selection to avoid imaging irrelevant parts of the sample. Beyond that, imaging may be restricted to specific objects that are of interest to a user, such as the dendritic tree of a specific neuron. For such applications, *SBEMimage* provides a framework to detect these features in real-time during acquisitions or to exclude images in which the existence of these features can be ruled out. Such inspection algorithms can be easily incorporated in *SBEMimage*’s acquisition cycle to select a given tile for registration or to discard it.

## Author Contributions

BT developed the software. CG performed beta-testing and provided detailed feedback and suggestions. BT and RF wrote the manuscript.

## Conflict of Interest Statement

The authors declare that the research was conducted in the absence of any commercial or financial relationships that could be construed as a potential conflict of interest.

## Footnotes

1 https://github.com/SBEMimage

2 https://github.com/vime

3 https://github.com/SBEMimage

## Supplementary Material

FIGURE S1 **(A)** The viewport’s workspace, zoomed out to show a stub overview image that covers the entire motor range of the stage. **(B)** The stage axes form a coordinate system that is rotated and scaled with respect to the SEM coordinate system. *SBEMimage* provides a calibration routine. Note that the motors move on slightly curved paths; the straight stage axes shown are an approximation.

FIGURE S2 Folder structure of a *SBEMimage* dataset and naming conventions for overview and tile image files. All images are saved as 8-bit greyscale TIFFs.
