# Supplementary figures and images for: SBEMimage: Versatile Acquisition Control Software for Serial Block-Face Electron Microscopy
Source: Front Neural Circuits. 2018 Jul 31;12:54. doi: 10.3389/fncir.2018.00054 (PMC6079252; doi:10.3389/fncir.2018.00054)

Figure S1

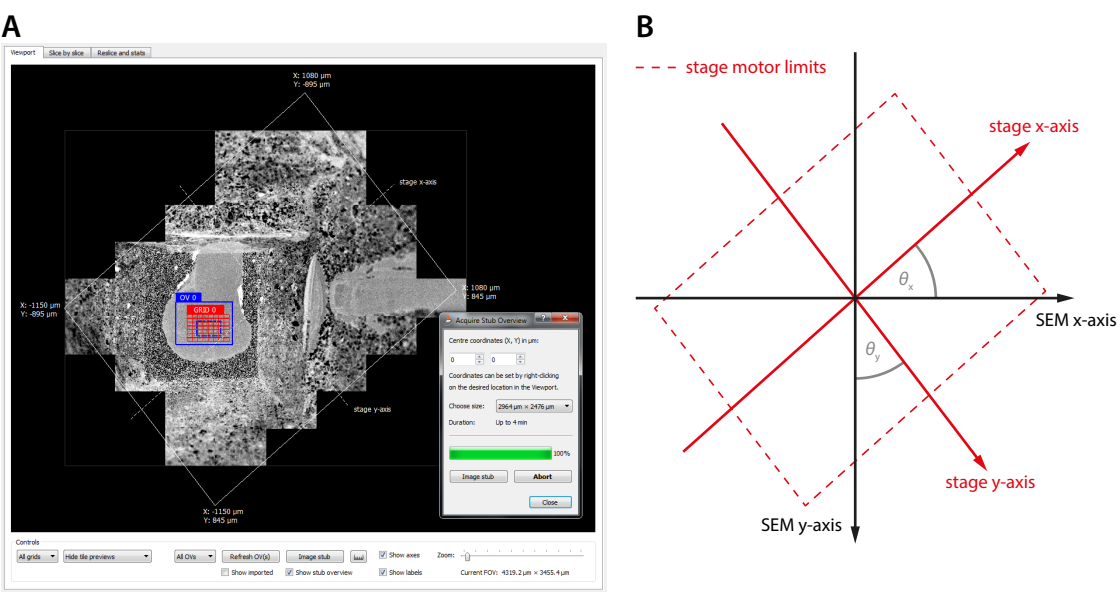

Figure S2

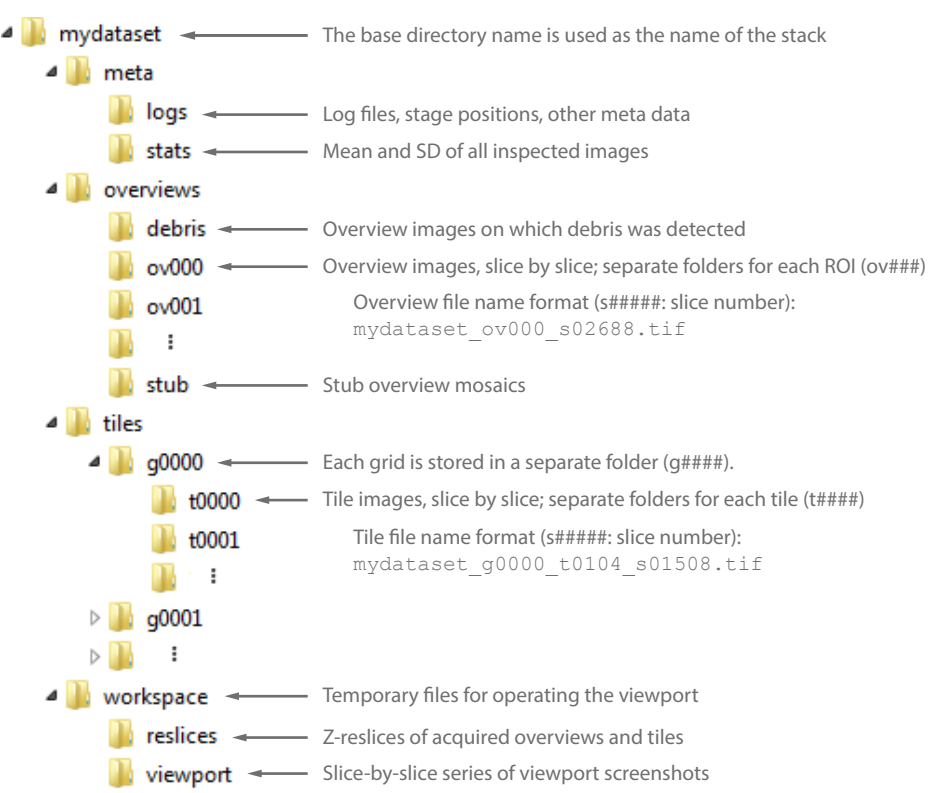

Supplement: FIGURE S1 — (A) The viewport’s workspace, zoomed out to show a stub overview image that covers the entire motor range of the stage. (B) The stage axes form a coordinate system that is rotated and scaled with respect to the SEM coordinate system. SBEMimage provides a calibration routine. Note that the motors move on slightly curved paths; the straight stage axes shown are an approximation. [file Image_1.pdf]
